# Prioritizing dumpsite risks and interventions: an overview and framework for action with a focus on LMICs

**DOI:** 10.1007/s10661-025-14842-5

**Published:** 2025-12-03

**Authors:** Giovanni Vinti, Valerie Bauza, Thomas Clasen, Daniele Di Trapani, Gaspare Viviani

**Affiliations:** 1 Deparment of Engineering, University of Palermo, Palermo, Italy; 2 Gangarosa Department of Environmental Health, Rollins School of Public Health, Emory University, Atlanta, GA USA; 3 The Aquaya Institute, San Anselmo, CA USA

**Keywords:** Dumpsites, Risk assessment, Comparative risk methodology, LMICs, Research direction

## Abstract

Dumpsites represent a major environmental and public health challenge, particularly in low- and middle-income countries (LMICs). Despite increasing global waste generation, many regions still struggle to implement safe and sustainable waste management practices, resulting in significant pollution, greenhouse gas emissions, and health risks due to uncontrolled waste disposal. Dumpsites lack containment measures, allowing pollutants to migrate through environmental matrices and reach different targets (e.g., human receptors). The primary objective of this review was to evaluate existing risk assessment procedures and propose a research direction for a standardized human health risk assessment (HHRA) methodology to prioritize interventions, starting with the most dangerous dumpsites in a given territory. The paper begins by presenting an overview of dumpsites, followed by a discussion on the pathways through which pollutants may migrate from dumpsites to human receptors and the associated health risks. Citing case studies, we explore the available environmental and health risk assessment methodologies, identifying and discussing key characteristics appropriate for dumpsites with a focus on LMICs where such sites are widespread and appropriate approaches are necessary. We identify and further discuss the relative HHRA as the most practical and reliable approach. We conclude with recommendations on research and policy.

**Supplementary Information:**

The online version contains supplementary material available at 10.1007/s10661-025-14842-5.

## Introduction

Unprecedented levels of solid waste generation globally represents one of the most fundamental challenges for our society and the planet (Lu et al., 2024). The quantity of solid waste produced has increased dramatically in recent decades, a trend that is expected to continue: between 2020 and 2050 municipal solid waste (MSW) generation per year is projected to grow from 2.1 billion tons to 3.8 billion tons, a 81% increase within a generation (UNEP, 2024). The great majority of solid waste is generated in high-income countries (HICs), especially in the United States (Statista, 2025). However, the amount of MSW in low- and middle-income countries (LMICs) has increased dramatically, from about 0.64 billion tons in 1970 to 2 billion tons in 2019. By 2050, the amount of waste in LMICs is predicted to increase by more than three times (Kaza et al., 2018).

In 2020, 38% of the overall MSW production (810 million tons) was uncontrolled: dumped in the environment or openly burned. If waste management practices remain the same as today, by 2050 this figure will almost double to 1.6 billion tons (UNEP, 2024). If not adequately managed, solid waste can pose significant threats to the environment and human health (Mazzucco et al., 2019; Vinti et al., 2024). When solid waste is disposed of in landfills and dumpsites, the organic fraction usually present in the waste undergoes anaerobic digestion, generating biogas, whose main components are two relevant greenhouse gases (GHGs), i.e. methane (CH_4_) and carbon dioxide (CO_2_) (Di Trapani et al., 2019). Even in well-managed landfills in HICs, such as Denmark, Sweden, and the United States, the reported average biogas collection efficiencies were only 50%, 58%, and 63%, respectively (Duan et al., 2022), while the rest was released into the atmosphere. At the same time, waste burning in dumpsites is a strong contributor of GHG emissions (Gómez-Sanabria et al., 2022). This process also causes the formation of highly polluted leachate, which can reach soil and groundwater, causing health risks.

Several factors influence the potential impact of solid waste on health. This makes it essential to consider the connections between potential sources of exposure from different waste management practices, environmental transport pathways of contaminants, and the possible adverse health outcomes (Vinti et al., 2023a). Dumpsites are characterised by not having any barriers or capping installed to prevent leachate and biogas dispersion, respectively (Lavagnolo et al., 2023). Therefore, contaminants can more easily impact human receptors and the environment, posing a higher risk compared to sanitary landfills.

In LMICs, open dumpsites often represent the most common waste management practice (UNEP, 2024; Villa et al., 2025), with limited management strategies implemented. The definition of dumpsites shares many similarities with that of “old landfills”, here referring to waste disposal sites, now closed and managed without proper legislation until a few decades ago in HICs. Indeed, even in Europe and United States it is only since the 1960 s that more controlled landfills have been made for waste disposal (Cossu, 2010). Thus, old landfills, as we coined here, should be viewed as a subset of dumpsites that were closed in HICs due to regulations update. Such an analogy could be seen as a game changer to adapt established strategies to LMICs. Indeed, the implementation of more restrictive regulations has led to a reduction in health risks within the source-pathway-receptor model. However, old landfills still exist. Consequently, the European Environment Agency (EEA) has collected information over the last few years about the relative risk analysis criteria that have been used to adequately prioritize and manage the interventions associated with contaminated lands (ISPRA, 2022).

However, a difference may be noted between old landfills and current dumpsites. The latter are often (but not always) in operation; thus, until such waste disposal sites are closed, specific operational activities aimed at immediate improvements should be implemented (UNEP, 2021), especially for sites presenting the highest risks. In the case of old landfills, except in instances of illegal management, the primary issue to address (after a risk assessment) is their remediation or securing to prevent the dispersion of pollutants.

Consequently, the high number of potentially contaminated dumpsites or old landfills and a general lack of funding, especially for the LMICs, require a globally shared approach. A comparative method to evaluate risks and prioritize the sites for intervention is essential. In this light the main objective of the present review is to assess existing risk procedures and propose research directions in terms of a standardized comparative risk methodology for dumpsites with particular attention to LMICs. Among the risk procedures investigated, the one identified as the most practical and accurate that will be discussed in more detail in Sect. "A comparative methodology for evaluating risks and prioritise the interventions in dumpsites", has already been applied in some HICs. The procedure is particularly well suited to many LMICs, where detailed information on the boundary conditions of dumpsites is often difficult to obtain. It can be implemented in old landfills of HICs when other measures and regulations have not already been taken.

In this paper, we begin by presenting an overview of dumpsites. We then discuss the source-pathway-receptor model associated with the environmental matrices and related health risks and present case studies evaluating health effects from dumpsite exposure. Next, we delve into the available environmental and health risk assessment methodologies used over the years. Building on previous approaches, we propose an optimized site-specific comparative method suitable for application to dumpsites, with particular focus on LMICs. The manuscript ends with conclusions and perspectives to address and suggest future research directions.

## Dumpsite regulation and practices

Solid waste generation can be defined as the unintended by-product of consumption and production; it is a direct consequence and a challenge associated with urbanization, economic development, and population growth (UNEP, 2024). Solid waste can be separated into two macro-categories (Kaza et al., 2018): (a) MSW, including residential, commercial and institutional waste, and (b) special waste, including industrial waste, construction and demolition waste (CDW), waste from electrical and electronic equipment (WEEE), and health-care waste, among others. Some categories of MSW and special waste can be classified as hazardous or non-hazardous depending on the origin or the concentration of some pollutants, but hazardous waste is usually more common in special waste.

The European Waste Framework Directive 2008/98 (European Commission, 2008) sets the basic concepts and definitions related to waste management. In particular, the waste hierarchy principle is presented, where the following order of priority is considered: waste prevention and reduction; reuse; recycling; energy recovery; and landfilling. Thus, waste disposal in landfills should be seen as the last resort, and waste dumping as a “non-solution”.

However, at the global level, about two-thirds of solid waste is disposed of in landfills and dumpsites (Kaza et al., 2018). Adequate waste management can be expensive and waste collection rates are influenced by income and the presence of urban or rural areas. For example, on average, urban and rural collection rates in HICs are about 100% and 98% respectively, while in low-income countries collection rates are about 48% and 28% respectively (Kaza et al., 2018). In Africa, about 90% of MSW is openly dumped or burned (Gómez-Sanabria et al., 2022).

In one of the few studies that focused on LMICs, Muheirwe et al. (2022) showed that in Sub-Saharan Africa, the relationship between formal regulation and solid waste management (SWM) performance was complex. The authors found that the mere existence of national or local regulations did not consistently predict improved SWM outcomes. They reported cases in which countries with inadequate policies (e.g., Swaziland) exhibited relatively strong waste-management performance, whereas countries with seemingly adequate legal frameworks (e.g., Ghana and Nigeria) continued to experience persistent problems. They also identified examples (e.g., Kenya and Tanzania) where coherent policy frameworks, when coupled with effective implementation, were associated with measurable improvements. They conclude that improving SWM is challenging and success depends on many factors such as adequate policy design, stakeholders’ participation and sustainable approach based on the local context.

In the last decade, both the International Solid Waste Association (ISWA, 2016) and the United Nations Environment Programme (UNEP, 2021) have published reports to track roadmaps for the progressive closure of dumpsites. ISWA focused on the 50 biggest dumpsites in the world, identified in LMICs, while UNEP concentrated on the Latin American and Caribbean dumpsites. Both reports highlight that accurate data about dumpsites can be difficult to ascertain. They also emphasize that the closure of dumpsites represents a highly challenging task that necessitates the implementation of a comprehensive integrated waste management strategy. Adequate technical capacity and financial resources are typically the main obstacles to enforcing such strategies (UNEP, 2021).

Identifying the location of dumpsites can sometimes be challenging for local authorities or environmental agencies. With this in mind, Sun and colleagues (Sun et al., 2023) have recently proposed using the novel pattern recognition method of deep convolutional networks applied to high-resolution satellite images to detect dumpsites. The authors detected about 1000 dumpsites in sampled areas of 28 cities from Africa, Asia, the Americas, and Europe, reducing the investigation time by more than 90% compared to a classical manual labelling method.

In HICs, solid waste legislation has improved only recently: although closely controlled now, solid waste was deliberately disposed of in uncontrolled landfills (i.e., dumpsites) until a few decades ago (Cossu, 2010). Nowadays, particularly in HICs, sanitary landfills must comply with very restrictive conditions, ensuring that the bottom lining, leachate collection system, and landfill gas collection system are adequately designed (Cossu & van der Sloot, 2014). Nevertheless, dumpsites have sometimes continued to be used even in European countries (Marfe & Di Stefano, 2016; Šedová, 2016). Additionally, what we defined as existing old landfills in HICs, although no longer in operation, in many cases have not been remediated, nor have they received a health risk assessment or safety works. For example, in Sicily (Southern Italy), 511 uncontrolled old landfills with suspected contamination were identified in the regional remediation plan (Sicilian Region, 2016). Depending on the source-pathway-receptor conditions, such sites can result in contaminated sites. In this scenario, over the last few years, the EEA has compiled information about the relative risk analysis criteria that have been implemented, and national strategies have recently been discussed, for instance, in Italy (ISPRA, 2022). Furthermore in HICs, waste disposal in dumpsites can be associated with illegal practices and organized crime interests (Baird et al., 2014; Troisi et al., 2024).

On the other hand, in many LMICs waste disposal in dumpsites is the most common practice. However, official censuses are very rare, particularly at the national level. Consequently, these contexts face a double challenge. Indeed, such waste disposal sites are in operation and, in many cases, officially allowed by local authorities (Diaz et al., 2007). Additionally, these sites are usually characterized by the lack of barriers or capping to prevent leachate and biogas dispersion (Lavagnolo et al., 2023; UNEP, 2021). Thus, as discussed in more detail below, they should be seen as sites with suspected contamination but with a challenging procedure to be proposed, to adequately manage and contain the risk. Dumpsites may differ depending on the urban or rural context; furthermore, in some cases only MSW are disposed of in them, while in other cases they mostly receive WEEE (WHO, 2021) or industrial waste from specific sectors. Additionally, mixed waste can be disposed of in them. In LMICs, the situation can be different. Indeed, waste disposal in dumpsites often has economic and technical reasons associated with everyday life (Villa et al., 2025). An ensemble of skills, technology, and financial constraints, alongside risk unawareness, leads to the perception of dumpsites and uncontrolled burning as the best waste management practices (Velis & Cook, 2021; Vinti & Vaccari, 2022).

**Fig. 1:**
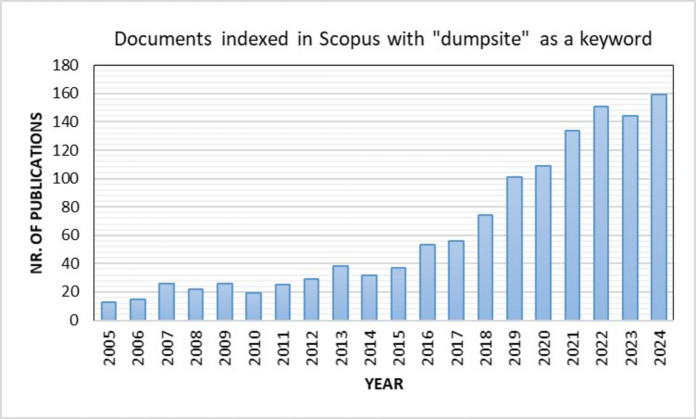
Documents indexed in Scopus from 2005 to 2024 using “dumpsite” as a keyword

In the context of dumpsites, scientific publications and research have been substantially increased over the last 20 years. A search in Scopus, one of the bibliographic databases most widely used by the scientific community, using the keyword “dumpsite” yielded the results shown in Fig. 1. As can be seen, the number of publications indexed in Scopus has grown more than tenfold over the past 20 years, rising from 13 in 2005 to 159 in 2024.

## Pollutant migration pathways

To unequivocally determine what should be conceived as a contaminated site may be challenging. Indeed, a contaminated site requires the following three conditions (Zhang et al., 2023): (i) source of contamination; (ii) one or more migration pathways for contaminants; and (iii) some targets (e.g., human receptors) along the migration pathways that are potentially threatened by contaminants. To define a site as effectively contaminated, the contamination threshold level for one or more pollutants in environmental compartments (water, air, soil), determined through a health risk assessment methodology (as discussed later), must be surpassed. This requirement is outlined in the recent proposal for a directive by the European Parliament on soil monitoring and resilience (European Commission, 2023). This approach is consistent with that already provided for by Italian regulations ( ISPRA, 2022).

**Fig. 2:**
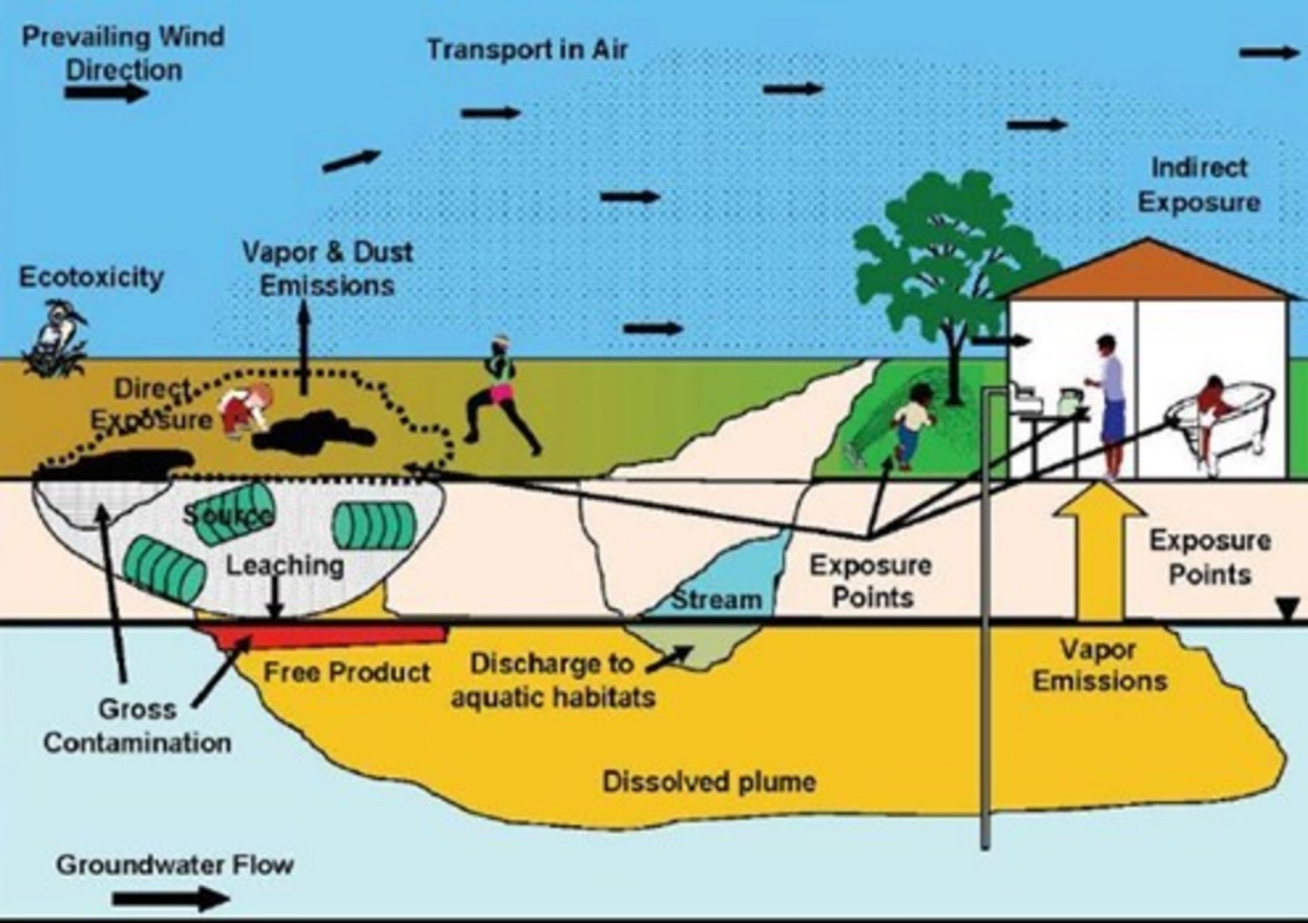
Potential exposure pathways from a dumpsite and human targets (reproduced with permission from ITRC (2012))

Thus, assessing the migration pathways is crucial and depends on the environmental matrices involved (Fig. 2). Infectious pathogens can be spread by vectors, such as insects or rodents, from waste (Krystosik et al., 2020), and biological risks should also be considered. Some authors have also assessed physical risks (e.g., injuries and burns) when evaluating the threat from dumpsites (Vinti et al., 2023a, 2024). Biomagnification and the accumulation of contaminants through the food chain represent another threat. Nevertheless, it must be noted that in classical health risk assessment, only toxic and carcinogenic risks from chemicals are considered (Gibellini & Vaccari, 2021). However, as discussed later, these other risks have been considered in some approaches (e.g. biological, microbiological, etc.). Furthermore, the European Directive proposal also aims to improve soil health and achieve a good ecological status (European Commission, 2023). Indeed, in other countries, the ecological risk is also considered. For example, the Netherlands employs a comprehensive, risk-based framework for managing contaminated sites, considering both human health and ecological protection (Swartjes et al., 2012).

Migration pathways can involve water bodies, sediment, soil and air. In the case of dumpsites, contamination can affect both groundwater and surface water. Contaminant migration can reach groundwater through leachate percolation, with contaminant concentrations decreasing due to filtration in the unsaturated zone, dispersion, and subsequent dilution in the groundwater (Vaccari et al., 2018). In this case, human receptors can come into contact with contaminants by inhaling contaminated vapours or drinking contaminated water or through the food chain (Han et al., 2016). Furthermore, untreated leachate can reach surface water receptors (Mekonnen et al., 2020). Leachate is a highly contaminated water that contains a diverse array of pollutants, including heavy metals, nitrogen compounds, as well as other toxic and emerging contaminants at different concentrations depending on many site-specific conditions (Qian et al., 2024; Vinti et al., 2023b). Among the emerging pollutants, scientists have identified PFAS, endocrine-disrupting compounds, pharmaceuticals, and personal care products (Qian et al., 2024). These substances pose significant risks to human health and the environment (Arman et al., 2021; Chen et al., 2015) and their removal is often challenging.

Surface water can also be affected by particulate matter deposition from the dumpsite during windy days. Additionally, due to the interplay between sediment and the aquatic environment, pollutants can be absorbed or released by sediment making it both a sink and a source of contamination to be taken into account (Russo Tiesi et al., 2025; Sulistyowati et al., 2023).

Soil is usually the first environmental matrix affected by dumpsites. Most dumpsites lack a geomembrane, putting the waste in direct contact with the soil. Consequently, pollutants can reach human receptors by different pathways, such as dermal contact. Inhalation or ingestion of soil particles represent further exposure pathways where children may be most at risk (Vinti et al., 2023a). Furthermore, plants and animals can uptake contaminants from the soil. Through the processes of bioaccumulation and biomagnification, these contaminants may ultimately pose a risk to human receptors via the food chain (Currier et al., 2020; Sabir et al., 2022).

Air pollution from dumpsites is often difficult to manage. Gaseous emissions can spread into the atmosphere, but they can also result from particulate matter spreading into the atmosphere. In both cases, a dilution effect can be identified (Elmi et al., 2021; Khan & Hassan, 2021) to evaluate exposure levels for nearby populations. Nevertheless, it is important to consider that air pollutants can travel long distances. Besides biogas, other health and environmental threats, such as PCBs and polycyclic aromatic hydrocarbons (PAHs), have been found in the atmosphere nearby landfills and dumpsites (Petrovic et al., 2018; Salami & Popoola, 2023). Air pollution also characterizes waste burning; in this case, besides inhalation, the ingestion risk should not be underestimated because some pollutants, such as dioxins, tend to deposit into the soil and then accumulate in the food chain (WHO, 2019).

Infectious diseases represent a further risk. Dumpsites can provide breeding grounds and food for animals (e.g. rodents) and insects (e.g. mosquitos) that spread vector-borne diseases (Krystosik et al., 2020). Runoff and leaching of contaminated water and leachate from dumpsites can spread bacterial pathogens (Addy et al., 2023).

Physical risks associated with dumpsites can be due to many factors. For instance, dump landslides, although rare, have killed dozens and sometimes over a hundred people at once (Lavigne et al., 2014; Zhan et al., 2018). Injuries associated with the movement of people in dumpsites or due to open fires represent a further risk (Vinti et al., 2023a). Musculoskeletal disorders are another physical risk that can affect informal waste workers operating in dumpsites in the long term (Bonini-Rocha et al., 2021).

**Fig. 3:**
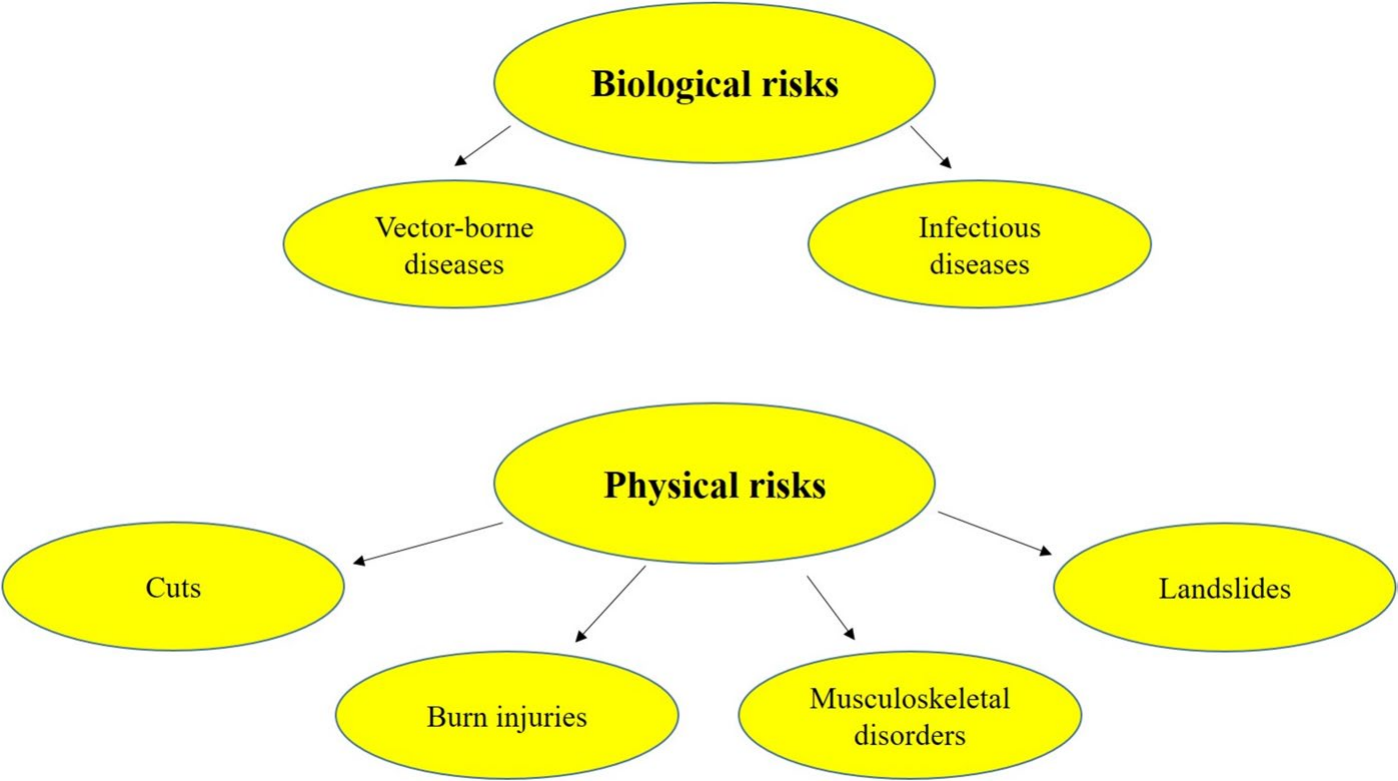
Examples of biological and physical risks associated with dumpsites

Examples of biological and physical risks associated with dumpsites are schematically summarised in Fig. 3.

## Health impacts from the literature: epidemiological and biomonitoring studies

Epidemiological and biomonitoring exposure studies provide evidence of health effects associated with landfill and dumpsite exposure, supplementing risk analyses and fate and transport models that predict the impact of waste disposal on human health. Epidemiology is a scientific discipline focused on the incidence and distribution of diseases within populations. Its primary objective is to identify factors or exposures that elucidate disease patterns and inform strategies for effective treatment and control. The field encompasses both descriptive and analytical domains, with a strong emphasis on etiological research (Laake et al., 2015). Biomonitoring studies can also be incorporated in epidemiological studies and represent another aspect of environmental health assessment. They utilize a range of tools and methodologies to measure and analyze the presence of biological markers in individuals or populations. By employing techniques such as blood or tissue sampling, biomonitoring offers valuable insights into the exposure levels of contaminants and other environmental agents (Izah et al., 2024).

Epidemiological studies and biomonitoring studies that investigate exposure to dumpsites are summarised in Table 1. It is noteworthy, however, these studies often have some limitations, as it is often challenging to conduct rigorous epidemiological studies in LMICs due to the frequent lack of adequate technical and economic resources, as well as the absence of medical facilities with detailed records to enable more rigorous health investigations beyond populations interviews.

**Table 1:** Summary of epidemiological and biomonitoring studies investigating the health effects of residing nearby dumpsites

Study location	Study design	Study population	Main findings	References
***Epidemiological studies***				
Ghana	Cross-sectional study	150 people living in a community nearby dumpsites, comparing three distances between people and dumpsites: (a) less than 5 min, (b) 5–10 min, (c) 11–15 min	Diseases which affected residents: - Cholera: (a) 67%; (b) 33%; (c) 0% - Typhoid fever: (a) 75%; (b) 25%; (c) 0% - Malaria: (a) 73%; (b) 25%; (c) 2%	(Suleman et al., 2015; Vinti et al., 2021)
India	Cross-sectional study	Exposed population: 200 people staying within a radius of 1 km of a dumpsite for at least 1 year Non-exposed (control) population: 200 people from a distant community having similar socio-economic and living conditions	The study found a relatively higher prevalence of selected morbidities among the exposed group than non-exposed group: - Respiratory illness (23% vs 10%) - Eye irritation (20% vs 9.5%) - Stomach problem (27% vs 20%)	(Singh et al., 2021)
Malaysia	Cross-sectional study	The exposed group included 170 residents within a 1 km radius and the non-exposed group (control) included 119 residents between a 2.5 and 4.0 km radius from a dumpsite	Dumpsite exposure was significantly associated with sore throat; diabetes mellitus; and hypertension	(Norsa’adah et al., 2020)
Nigeria	Cross-sectional study	100 households living within 250 m radius of a dumpsite and 100 households living between 250–500 m from the same dumpsite	Diseases which affected residents: - Cholera and diarrhea: 10 households closer to the dumpsite reported 1–2 cases, compared to 5 households further away. No households, either closer or further away, reported more than 2 cases -Malaria: 20 households closer to the dumpsite reported 1–2 cases, compared to 24 households further away. 4 households closer to the dumpsite reported 3–4 cases, compared to 8 households further away. 0 households closer to the dumpsite reported at least 5 cases, compared to 1 household further away	(Babs-Shomoye & Kabir, 2016; Vinti et al., 2021)
Nigeria	Cross-sectional study	81 people living and 89 people working within 1 km of a dumpsite. Three periods of living or working in the study area were considered: 0–5 years, 6–10 years, more than 10 years	The duration of residence was associated with the frequency of symptoms, with higher odds of self-reported daily symptoms for individuals who had been living or working in the exposed community for 6–10 years or more than 10 years, compared to those who had resided there for 0–5 years In particular, significantly increased symptoms were observed (for residence duration above 10 years) for: Headaches; whiteness of fingers; confusion; memory problems; extreme fatigue; insomnia; tremor/cramps; joint problems; backache	(Adetona et al., 2020)
Sierra Leone	Cross-sectional study	398 residents living within 50 m of the dumpsite and 233 residents living beyond 50 m from the dumpsite	Diseases which affected residents: - Diarrhea: Approximately 10% of residents living closer to the dumpsite compared to approximately 12% of residents living further away - Cholera: Approximately 11% of residents living closer to the dumpsite compared to approximately 15% of residents living further away - Malaria: Approximately 45% of residents living closer to the dumpsite compared to approximately 35% of residents living further away	(Sankoh et al., 2013; Vinti et al., 2021)
Swaziland	Cross-sectional study	78 residents living in close proximity to a dumpsite, and 78 residents divided into two groups: 39 living within 200 m and 39 living beyond 200 m from the dumpsite	Diseases which affected residents: - Diarrhoea: 16% of residents living closer to the dumpsite compared to 5% of residents living further away - Malaria: 36% of residents living closer to the dumpsite compared to 13% of residents living further away	(Abul, 2010; Vinti et al., 2021)
Zimbabwe	Cross-sectional study	194 people living within 90 m from dumpsites vs 286 people living far away	A higher prevalence of diarrheal diseases among people that lived within 90 m from the dumpsites than those who lived outside these perimeters (84 vs 63 people)	(Khumalo et al., 2021)
***Biomonitoring studies***				
Ghana	Cross-sectional study	Exposed group: 327 people living or working around the Agbogbloshie WEEE dumpsite. This included 24 WEEE burners, 23 WEEE collectors, 23 WEEE dismantlers, 18 sorters, and 21 women market sellers, 137 worked in the local market, 24 worked in the factory nearby (located downwind from the dumpsite), and 57 school children from the neighboring school Control group: no external control group was recruited. Instead, the results were compared against European reference values (from Germany), which represents a limitation of the study	Burners (11.83 μg/l), dismantlers (9.5 μg/l), collectors (8.66 μg/l) and sorters (7.79 μg/l) exhibited the highest blood lead levels (BLL) among all groups A total of 18 men and 1 child had BLL exceeding 150 µg/L, i.e., above the German occupational reference value for individuals working with lead, which was used as a benchmark by the authors	(Püschel et al., 2024)
Italy	Cross-sectional study	Exposed group: 32 pregnant women from the area of the “Triangle of Death” (an area in Campania region, Italy highly polluted due to numerous illegal dumpsites) Control group: 6 pregnant women from Puglia region, Italy The serum levels of 12 polychlorinated biphenyls (PCBs) in maternal blood serum and umbilical cord blood serum	The PCBs levels were significantly higher in the exposed group not only compared to those found in the control group but also in comparison to those reported in other published studies	(Grumetto et al., 2015)
Kenya	Cross-sectional study	Exposed group: blood samples from pigs (2), goats (53), sheep (3), and cattle (5) were collected in Dandora dumpsite Non-exposed (control) group: blood samples from pigs (2), goats (33), and sheep (41) were collected around Lake Nakuru National Park	Blood Pb level (Pb-B) of goats and sheep from Dandora was significantly higher than those from Nakuru. Likewise, pigs from Dandora accumulated a high Pb-B level than those from Nakuru, although a significance test was not conducted because of the small sample size	(Nakata et al., 2015)
Nigeria	Cross-sectional study	Concentration, contamination levels, and associated health risks of heavy metals in ten leafy vegetable samples from two indigenous species (Amaranthus viridis and Talinum triangulare) grown at five dumpsites in Nigeria	Over 50% of samples reached an unacceptable carcinogenic risk level for ingestion, with incremental lifetime cancer risk values above 10^–6^ Cd posed the highest lifetime cancer risk for children (1.064 × 10^–3^)	(Otugboyega et al., 2024)
Zambia	Cross-sectional study	The study assessed the exposure of heavy metals to the population living near mine dumpsite Human samples: hair and nails Environmental samples: vegetables (collected from the most commonly consumed vegetables in the area); drinking water (from 5 wells)	The inhabitants had high health risk due to Cd, Mn, Ni, and Pb ingestion from vegetables (estimated values above the tolerable daily intake limits) and due to Cu, Mn, and Ni ingestion from drinking water Positive correlations between hair and nails as biomarkers of exposure and the dietary intake of vegetables and drinking water indicated a link between contamination of agricultural fields and groundwater supplies	(Nakaona et al., 2020)

### Epidemiological studies

In 2021, Vinti and colleagues (Vinti et al., 2021) conducted a systematic review that synthesized research in order to shed light on the adverse health outcomes associated with the presence of dumpsites and uncontrolled waste burning. The review found evidence suggesting people living near dumpsites have an increased risk of developing adverse birth and neonatal outcomes. Cross-sectional studies conducted in Ghana (Suleman et al., 2015), Nigeria (Babs-Shomoye & Kabir, 2016), Sierra Leone (Sankoh et al., 2013), and Swaziland (Abul, 2010) suggested living near dumpsites may be associated with an increase in gastroenteritis and malaria (Vinti et al., 2021), although the results were not statistically significant and opposite findings were found for malaria in Nigeria and gastroenteritis in Sierra Leone (see Table 1).

A cross-sectional study carried out in Nigeria (Adetona et al., 2020) investigated the associations between potential exposure to MSW combustion-related air pollution and adverse health symptoms among residents of a community adjacent to a large dumpsite in Lagos. The authors found that increased risk of exposure to combustion-related emissions from the dumpsite for long-term nearby residents, was associated with higher frequencies of respiratory, neurological, and musculoskeletal symptoms.

Another cross-sectional study (Norsa’adah et al., 2020) compared the prevalence of health symptoms and diseases among residents living near an open solid waste dumpsite in the suburb of Sabak, Malaysia, with those in a non-exposed community. The exposed group consisted of individuals residing within a 1 km radius of the dumpsite, while the non-exposed group included those living between 2.5 and 4.0 km away. The findings revealed a significant association between dumpsite exposure and certain health conditions, i.e. sore throat, diabetes mellitus, and hypertension (Norsa’adah et al., 2020).

Khumalo and colleagues (Khumalo et al., 2021) investigated the spatial distribution of illegal dumpsites and the association between living in close proximity to the sites with the prevalence of diarrheal diseases among residents in Bulawayo, Zimbabwe. A cross-sectional survey was conducted among individuals who had resided in Makokoba for at least six months prior to data collection. The findings indicated a higher prevalence of diarrheal diseases among residents living within a 90-m radius of the dumpsites compared to those residing beyond this distance (Khumalo et al., 2021).

Another cross-sectional survey conducted in Mumbai, India (Singh et al., 2021) used a propensity score matching approach with exposed and non-exposed groups to investigate health effects of living near dumpsites. Cases of the exposed population included people residing within a 1 km radius of the dumpsite for at least one year. Participants from a distant community, characterized by homogeneous socio-economic status and living conditions, were designated as the non-exposed group. The findings indicated a higher prevalence of adverse health outcomes among the exposed group compared to the non-exposed group, particularly for respiratory illness, eye irritation, and gastrointestinal problems.

### Biomonitoring studies

In addition to studies measuring health outcomes, biomonitoring studies were also conducted to better elucidate the complex interplay of interacting variables, their roles, impacts, and mitigation strategies. Biomonitoring strategies are useful in LMICs to investigate the intricate dynamics between emerging pollutants from urbanization and industrialization, and the consequences of food and industrial waste, agricultural activities, population growth, and natural and anthropogenic hazards (Izah et al., 2024).

In one of such studies (Nakaona et al., 2020), manganese (Mn), cobalt (Co), copper (Cu), lead (Pb), nickel (Ni) and zinc (Zn) were analysed in human hair and nails, locally grown vegetables, and drinking water to assess exposure levels resulting from dust transference via wind and rain erosion among residents of the Mugala community (Zambia), located near a mine dumpsite in the Zambian Copperbelt. The findings suggested that inhabitants were at risk of exposure to Pb, Ni, and Cd through the ingestion of contaminated vegetables and drinking water.

Considering that the conversion of dumpsites into farmland is a common practice in several communities across Nigeria, Otugboyega and colleagues (Otugboyega et al., 2024) evaluated the concentration, contamination levels, and associated health risks of heavy metals in two widely consumed indigenous leafy green vegetables, Amaranthus viridis and Talinum triangulare, cultivated on five major dumpsites in Lagos State. The findings highlighted high exposure levels of certain heavy metals from consuming vegetables grown on four of the five dumpsites studied, with children being the most vulnerable population for risk of carcinogenicity from long-term exposure.

Nakata and colleagues studied a large waste dumping site named Dandora, located Nairobi, Kenya (Nakata et al., 2015). The area is also a high-density slum, home to approximately 250,000 residents. The study assessed the contamination levels of metals and metalloids in the blood of pigs, goats, sheep, and cattle from Dandora. Elevated levels of Cd and Pb were found in blood samples, suggesting human exposure to heavy metals through livestock consumption. However, Cd concentrations in animals from the dumpsite and national park were comparable, requiring further investigations. Analysis of Pb isotope ratios indicated species-specific differences in exposure routes. Another study (Grumetto et al., 2015) was conducted in Southern Italy in a highly polluted area due to the presence of numerous illegal waste disposal sites. To evaluate whether environmental contamination contributes to increased exposure to harmful chemicals, serum levels of 12 PCBs classified as dioxin-like were measured in both maternal blood serum and umbilical cord blood serum of residents in the affected area. The results revealed significantly higher PCB levels compared to Italian people residing in other areas.

WEEE constitutes another category of waste that is often disposed of in informally designated areas in LMICs. While these sites serve as sources of valuable material recovery, they also frequently evolve into highly contaminated dumpsites (Vaccari et al., 2019). Consequently, several studies have reported adverse impacts on soil, water, and human health (Bhardwaj et al., 2025; Vaccari et al., 2019). Accordingly, biomonitoring studies have been conducted. In particular, Püschel et al. (2024) assessed lead exposure among individuals living and working at the Agbogbloshie WEEE dumpsite in Ghana, measuring blood lead levels (BLL) in 327 volunteers. Waste workers exhibited the highest BLL; however, other categories of residents also showed elevated levels, indicative of significant environmental exposure. Importantly, the study did not recruit an external control group from other regions of Ghana, but instead compared results with European reference values. This approach probably represents the study major limitation that future research should address. 

Overall, the results are consistent with the predictions of fate and transport models, considering the hazardous nature of substances typically found in dumpsites and the previously described risk of infectious disease spread. Indeed, the studies confirm a stronger correlation between adverse health outcomes and individuals residing near dumpsites compared to control groups.

## Health and environmental risk assessment

Over the years, many procedures have been proposed and implemented to quantify the risk posed by waste disposal in landfills and dumpsites. Estimating risk is necessary, as it is often not feasible to directly measure the health effects of landfills and dumpsites. The procedures identified are summarised in Table 2 and discussed hereinafter.

**Table 2:** Summary of health and environmental risk assessment methods for landfills and dumpsites

Name of method	Context of implementation	Method inputs	Outputs	Strengths	Limitations	References
Leachate Pollution Index (LPI)	Ethiopia, UK, etc	Only 18 leachate-related parameters	It quantifies the leachate contamination potential as LPI	It works also with less than 18 parameters	Site-generic (no other boundary conditions are considered; thus the source-pathway-receptor model is not considered) Human health risks are not considered	(Chekole et al., 2025; Kumar & Alappat, 2005)
Geo-accumulation Index (Igeo)	Worldwide	Concentration of the heavy metal in the soil sample and background value of the heavy metal in the region	It estimates the contamination levels of heavy metal contents in soil above the background level	It provides a quantitative measure of heavy metal contamination with a standardized classification It is effective in distinguishing between natural and anthropogenic contamination	It only focuses on ecological risks. Thus, human health risks are not considered If no background values are available, the uncertainties can be excessive	(Afolagboye et al., 2020; Andaloussi et al., 2025; Choudhury et al., 2022)
Enrichment Factor (EF)	Worldwide	Concentration of heavy metal in the environmental matrix considered (e.g., air, soil, sediment) Background value	Enrichment factor classification to quantify heavy metal inputs above natural levels	It provides a quantitative measure of heavy metal contamination with a standardized classification It is effective in distinguishing between natural and anthropogenic contamination	It only focuses on ecological risks. Thus, human health risks are not considered	(Afolagboye et al., 2020)
Municipal Risk Index (MRI)	Italy	Location of waste disposal site (with institutional databases and GIS); site characteristics (e.g., waste type, disposal method)	MRI categorized into four risk classes	Applicability in data-scarce contexts	Lack of detailed environmental data (the analysis does not consider direct quantification of chemical contaminants; risk assessment is inferred from the classification of waste and disposal methodologies)	(Fazzo et al., 2020)
Multi-criteria spatial modelling	Ethiopia	Field-collected data on dumpsites and groundwater well points using GPS Interviews with residents Satellite images	Multi-criteria decision analysis using analytic hierarchy process for weighted factor evaluation Weighted linear combination to generate suitability maps Constraints analysis (e.g., proximity to water sources and residential areas) to exclude unsuitable locations	Efficient decision-making (the GIS-based methodology facilitates accelerated analysis and identification of optimal waste disposal site locations)	Method used for identify suitable waste disposal site locations. Not to evaluate the risk associated to existing dumpsites Data Availability (secondary data sources are used, which may introduce potential biases due to data limitations and accuracy constraints.) Lack of Direct Pollution Assessment	(Mussa & Suryabhagavan, 2021)
Hazard Ranking System (HRS)	USA	The system relies on preliminary site assessments rather than detailed field investigations (input data based on the source-pathway-receptor model) Sources include historical records, preliminary site assessment	Hazard Score (0–100) Risk through groundwater, surface water, air, and soil exposure pathways	It assesses multiple contamination pathways	The scoring system is highly dependent on input parameter accuracy, which can vary due to data gaps The ranking score may increase by growing the quantity and quality of supporting information; however, this could result in potential misinterpretation	(US Epa, 2024)
Blacksmith Index	LMICs	The source and scale of pollution The size of the affected population The exposure (e.g., ingestion, inhalation, dermal contact)	Risk ranking score (0 to 10) for each site Identification of priority sites for intervention based on human health risk	Cost-effective and time-efficient Adaptability for LMICs	It does not assess potential future contaminant migration from the contaminated site to human receptors Lack of accuracy and possible underestimation	(Caravanos et al., 2014)
Human health risk assessment (HHRA)	Australia, Canada, China, Europe, USA	Hazard Identification; Dose–Response Assessment; Exposure Assessment; Risk Characterization	Risk values indicating potential health hazard (in terms of carcinogenic or toxic risk)	Covers all aspects of exposure, from contaminant source to human receptor Flexibility (it is applicable to different environmental matrices and pollutants)	High-level of site-specific data required (possible resource constraints, particularly in LMICs)	(Zhang et al., 2023)
Relative risk analysis (relative HHRA)	European countries	Data on contamination sources, pathways, and receptors	A prioritized ranking of contaminated or potentially contaminated sites (including dumpsites) based on their relative risks	It can be applied with limited information, using default values where site-specific data are unavailable Adaptable to varying levels of data availability	Default values can lead to uncertainties	(ISPRA, 2022, 2023)
Risk Screening Method (RSM)	China	Existing data and site inspection rather than extensive field sampling 15 soil risk indicators and 14 groundwater risk indicators	A total risk score (from 15.7 to 100.0), combining soil and groundwater risk scores	It can be applied to a lot of sites using minimal site-specific data Cost-effective	Limited accuracy It does not account for long-term pollutant migration dynamics For its implementation in dumpsites updates or changes appear to be necessary	(Li et al., 2021)
Risk-based corrective action (RBCA)	USA	Chemical concentration in the environmental matrices, exposure pathways, site-specific conditions and receptor characteristics	Risk estimates, Risk-Based Screening Levels (Tier 1), Site-Specific Levels (Tiers 2 and 3), remediation action plan	Flexibility (implementable both in simple and complex sites), cost-effective, combination of risk assessment and corrective actions, tool support (i.e. software)	In contexts with limited data availability (e.g., dumpsites in LMCIs), adequate implementation can be challenging Specialized expertise in risk assessment, contaminant transport modeling, toxicological analysis, software use is necessary	(Connor & Newell, 1999)
Regional risk assessment (RRA)	Poland	Data about contamination sources (e.g., type, toxicity, extent, and duration of polluting activities) Environmental parameters (GIS)-based spatial datasets	A ranked list of potentially contaminated sites based on estimated relative risks	It uses a structured, multi-criteria decision analysis approach Adaptable to different regional contexts	It requires detailed and accurate spatial and contamination data It relies on certain assumptions regarding contaminant behaviour that may introduce uncertainties	(Pizzol et al., 2011)
Solid Waste Safety Plan (SWSP)	Ghana, Serbia	Data Collection (i.e., interviews with local stakeholders, field and satellite observations, technical and scientific data), hazards identification	Health risk assessment matrix Control measures to mitigate the risks Cost analysis for control measures implementation	It considers multiple SWM practices and associated health risks It can be adapted to different contexts and data availability The presence of control measures and economic analysis	Due its novelty, it requires further investigations and to be tested with other case studies Some risk assessments can rely on expert judgment and literature data in case of lack of local data	(Vinti et al., 2023a, 2024)

Kumar and Alappat (2005) introduced the Leachate Pollution Index (LPI), a quantitative tool for reporting the pollution data quantifying the leachate contamination potential. The LPI includes 18 parameters, such as heavy metals, organic and inorganic pollutants. Additionally, it can be adopted even if data is not available for all 18 parameters. However, the index is site-generic and does not consider the migration pathways, the human receptor exposure, and health risks. It implies that the presence of a groundwater or residents nearby does not influence the risk and further tools should also be integrated (Chekole et al., 2025). Similarly, some authors have focused on ecological risk assessment using other indices, such as the Geo-accumulation Index (Igeo) and Enrichment Factor (EF) (Afolagboye et al., 2020; Andaloussi et al., 2025; Choudhury et al., 2022). These two indices, like other ecological ones, evaluate the impact of human activities on the environment. However, they do not consider or infer health risks.

Other authors have proposed a Geographic Information System (GIS)-based approach to assess the potential risk posed by dumpsites and landfills. For example, Fazzo and colleagues (Fazzo et al., 2020) used GIS to estimate the waste risk exposure in an area with illegal dumpsites and burning sites in Italy. The authors computed a Municipal Risk Index (MRI), i.e., a GIS-based indicator of the waste risk considering type and quantity of waste, nature of disposal sites, and proximity to inhabitants and the municipalities. Another GIS-based approach consisted of a multi-criteria spatial modelling (Mussa & Suryabhagavan, 2021); however, it was used for the selection of suitable dumpsite locations in Ethiopia. In particular, the authors based their study on thematic maps (e.g., groundwater well, road network, river, land-use and land-cover, soil map). The suitability map was then prepared using overlay analysis, categorizing areas as highly suitable, suitable, moderately suitable, less suitable, and unsuitable. The method implemented in Ethiopia could potentially be used inversely to create a hazard map of the various dumpsites in a given geographical area.

Another approach uses the human health risk assessment (HHRA). It is based on hazard identification, dose–response assessment, exposure examination, and risk characterization. However, it also requires a lot of detailed site-specific information. Based on the HHRA guidelines and standards have been enforced in many Industrialised Countries, such as in Australia, Canada, China, European countries, and the USA (Zhang et al., 2023). In Italy, there is a procedure that has also been implemented for landfills risk evaluation (Gibellini & Vaccari, 2021). However, given the required high-level of site-specific data, the implementation of a classical HHRA could be challenging in many LMICs.

The identification of several contaminated sites within a given region has led to the introduction of relative risk analysis criteria to rank risks and prioritize sites for further investigation or earlier remediation actions (Pizzol et al., 2011). US EPA introduced such a procedure in the early 1980 s, with the Hazard Ranking System (HRS), where to facilitate the ranking process, it was not necessary during this first step to calculate an absolute level of risk (i.e., the carcinogenic or toxic risk posed by the site was not measured) (Haness & Warwick, 1991). Since then, the system was revised. Under the HRS, four pathways can be evaluated (US Epa, 2024): (1) Groundwater migration (drinking water); (2) Surface water migration (drinking water, human food chain, sensitive environments); (3) Soil exposure and subsurface intrusion (population, sensitive environments); (4) Air migration (population, sensitive environments). However, the hazard ranking system scores do not determine the priority in funding EPA remedial response actions (US Epa, 2024), but a site with a score of 28.50 or higher out of 100 will be eligible for further Superfund processes and investigations, whereas a site with a score below 28.50 should receive a “no further remedial action planned” recommendation (Sha, 2014). However, the system has received some criticism over the years. For example, the ranking score can increase when enlarging the amount and quality of the support information, but it could lead to misinterpretation (Gottinger, 1997; Haness & Warwick, 1991); furthermore, the system places excessive emphasis on the surface water pathway (Bergius & Öberg, 2007).

A further approach is the Risk-Based Corrective Action (RBCA) developed in the United States in the 1990 s (Connor & Newell, 1999). It is a systematic decision-making process that integrates site-specific risk assessments with corrective action strategies. RBCA utilizes a hierarchical methodology: Tier 1 applies conservative and site-generic screening criteria, while Tiers 2 and 3 incorporate progressively more site-specific data and modelling to establish remediation objectives. However, in contexts with limited data availability (such as in many LMCIs), application can be challenging or unreliable. Additionally, proper implementation requires specialized expertise in risk assessment, contaminant transport modelling, and toxicological analysis. Finally, personnel training for software use is often necessary.

Sing and colleagues (Singh et al., 2010) reviewed 18 groundwater contamination hazard rating systems investigating if they could be appropriate for dumpsites taken as a reference. In general, the systems did not perform well due to inadequate sensitivity to MSW characteristics often emphasizing excessively factors such as mobility and toxicity. Among them, only the US EPA HRS previously mentioned showed some promising results, although shortcomings were noted analogues to those above mentioned.

To overcome excessive detailed information that can be needed, Caravanos and colleagues (Caravanos et al., 2014) suggested to use the Blacksmith Index, i.e. a simplified risk-ranking system for prioritizing potentially contaminated sites in LMICs. However, the Blacksmith Index only focused on the contamination at receptor areas; thus, the pollutants migration pathways from the dumpsite to potential human targets are not considered. Thus, as the authors highlighted, the Blacksmith Index misses the risk associated to potential future contamination and focuses only on current or past contamination at receptors.

European countries have also implemented relative risk analysis (ISPRA, 2022). It has been recently introduced in Italy for both contaminated and potentially contaminated lands, including old uncontrolled landfills, and a software named ROCKS was implemented for potentially contaminated lands (ISPRA, 2022, 2023). An additional relative risk approach has been adopted in some Italian regions, such as Sicily, for suspected contaminated sites (Sicilian Region, 2016), i.e. sites whose environmental matrices (e.g., soil or groundwater) have not been characterized (they did not receive chemical analysis and pollutants concentrations are unknown). Noteworthy, the approach implemented in Sicily goes beyond the weakness noted by Haness and Warwick (Haness & Warwick, 1991) in the HRS. Indeed, following a conservative approach, it gives strength to some unknown parameters (for example, when information about hydraulic conductivity or presence of wells nearby is unavailable) (Sicilian Region, 2016).

A similar approach developed in China is the Risk Screening Method (RSM) for industrial sites (Li et al., 2021). It consists of a scoring system composed of source-pathway-receptor indicators based on desk study and field inspections. Similar to the relative HHRA, it aims to prioritise potentially contaminated sites and decide the ones that deserve further investigation. The prioritization scoring matrix to be fulfilled has great potential (Li et al., 2021), but for the implementation in dumpsites, changes would be necessary, taking into account that the method was developed for closed or relocated industrial sites. Instead, the Italian relative HHRA considers parameters that are more specific for waste disposal sites (e.g., leachate, biogas or uncontrolled waste disposal).

Another relative risk approach was proposed by Pizzol and colleagues (Pizzol et al., 2011), known as the regional risk assessment (RRA) methodology. The authors split the methodology into six steps: (I) regional exposure diagram; (II) general relative risk model; (III) hazard analysis; (IV) vulnerability analysis; (V) pathway relevance analysis; (VI) regional risk estimation. The methodology could also be applied in waste disposal sites. However, its implementation appears to be challenging for dumpsites in LMICs since the methodology relies heavily on high-quality, comprehensive data.

A further approach that has recently been proposed consists of the Solid Waste Safety Plan (SWSP), implemented in Ghana (Vinti et al., 2023a) and Serbia (Vinti et al., 2024). The SWSP was modelled after consolidated safety plans already promoted by WHO in the field of drinking water (Davison et al., 2005) and sanitation (WHO, 2015, 2022). A crucial element was represented by the health risk assessment matrix, which allowed for comparing different hazardous events using a common yardstick. Completion of the risk assessment matrix allows users to evaluate different SWM activities and the related hazardous events (e.g., leaking of leachate from the dumpsite into groundwater, free movement of people or farm animals in the dumpsite, etc.). The method was useful for assessing SWM activities even when quantitative data were scarce and challenging to collect, and appropriate control measures to mitigate the highest risks were identified and discussed. Finally, a cost analysis was also presented in the Serbia case study (Vinti et al., 2024). However, given the novelty of the SWSP approach, it needs to be further consolidated.

## A comparative methodology for evaluating risks and prioritise the interventions in dumpsites

Among the methodologies discussed in the previous section, we identified the relative HHRA and the SWSP as the most practical and accurate that could be implemented particularly in LMICs. Dumpsites are common in LMICs, but detailed information can be challenging to collect. The SWSP was recently implemented in rural areas of Ghana (Vinti et al., 2023a). However, the SWSP deserves further validation since it has been theorized and implemented only over the last few years; thus, as authors highlighted (Vinti et al., 2024), it requires further investigations and to be tested with other case studies for refining its structure. Consequently, we finally focused exclusively on the relative HHRA, which is further discussed below.

In the relative risk procedure, three macro-categories of sites can be distinguished: contaminated sites, potentially contaminated sites, and suspected contaminated sites. Taking the Italian context as a reference, they can be defined as follows (ISPRA, 2023; Sicilian Region, 2016):

- Contaminated sites: areas where a HHRA has been conducted, and the contamination is confirmed.
- Potentially contaminated sites: a first characterisation has been conducted; concentration is known and some contamination threshold concentrations have been exceeded in the environmental matrices. The site requires a HHRA to confirm the contamination and the related human health risks.
- Suspected contaminated sites: Information about the concentration of pollutants in the environmental matrices is not known. However, activities that are or were conducted in the site may have caused its contamination.

However, the definitions of potentially and suspected contaminated sites can vary among countries, and sometimes, they are categorized together. For instance, European institutions (EEA, 2024; European Commission, 2023) consider potentially contaminated sites as areas where both contamination and associated risks are “suspected”, thus they are included in the same category.

A relative risk analysis for what we defined as old landfills has already been successfully implemented in Italy and could represent a good starting point; indeed, such old landfills were investigated as suspected contaminated sites. Thus, field visits without technological devices could be sufficient to complete most of the related technical data sheets required for the assessment (Table S1 and Table S2 in Supplementary Materials,). Additional information could be collected using freeware mapping software such as Google Earth.

Information from all the dumpsites in the territory involved (e.g. at the regional or national level) should be summarized in a table (Table S1). The sites with the highest risk values should be prioritized and the contamination threshold concentrations should be measured in each potentially contaminated environmental matrix. Depending on financial and technical availability at the national or regional level, the contamination threshold concentrations could be measured only in a few of such sites. Groundwater quality should be monitored upstream and downstream of the dumpsites where more people are potentially exposed, for example using the Italian legislation on sanitary landfills as a reference (Italian Legislative Decree, 2003). Dumpsites where contamination threshold concentrations limits are exceeded should undergo a second level of relative risk investigation aimed at completing a more detailed technical data sheet, using the one developed by ISPRA (ISPRA, 2023) for potentially contaminated sites as a reference (see Table S2, Supplementary Materials). In this step, more detailed information will be required to characterize the waste site and proximity to affected areas. Resulting information from these dumpsites should be summarized in a second table. The sites with the highest values should be prioritized and receive a HHRA. Also in this case, the number of dumpsites that will receive the HHRA will depend on financial and technical availability at the national or regional level. For this reason, the ranking system characterising the relative HHRA plays a pivotal role.

At this stage, a risk analysis should be conducted for all relevant sites. Given the limited financial and technical resources in many LMICs, it is unlikely that extensive site characterizations and multiple HHRAs will be performed. Remediation strategies should be conceived immediately for sites identified as contaminated following the HHRA.

However, in the rare case that multiple HHRAs are conducted, and a large number of contaminated sites are identified, a third-level comparative risk assessment should be carried out for all contaminated sites. This would allow for the prioritization of interventions based on relative risk. In this case, there would ideally be another technical data sheet to compare the relative risk posed by each contaminated site and prioritise the interventions. However, as of January 2025, such third level technical data sheet was not definitive yet in Italy. Thus, while waiting for updates that could arrive over the next few years at the Italian or European level, operational safety works should be conceived in all the sites to reduce the risks identified. Biological risks in all sites should also be reduced, starting with installing fencing around the dumpsites.

## Remarks on long-term solutions

Long-term solutions, such as remediation interventions, typically require substantial time and significant financial resources. Meanwhile, actions for the immediate improvement of dumpsites conditions should be implemented. Such improvements should aim to reduce leachate generation or collect it, reduce air pollution (e.g., hindering open burning of waste), and prevent people from having direct contact with waste (e.g., by means of fences) (UNEP, 2021; Vinti et al., 2024). For instance, in LMICs, appropriate leachate treatment can be achieved by the use of constructed wetlands (Bakhshoodeh et al., 2020) although detailed site-specific information will be necessary.

It is important to consider that if monitoring of wells upstream and downstream of the dumpsite demonstrates drinking water contamination, rapid interventions will be required in order to reduce the health risk for people as soon as possible. Depending on the contamination, intervention at the point of use should be implemented; otherwise, water consumption from the contaminated wells should be discouraged.

The remediation strategies will depend on financial availability as well as the kind of contamination. For instance, in the case of groundwater contamination, a permeable reactive barrier (PRB) could embody the best remediation solution. Indeed, PRB represents a passive and sustainable technology for the in-situ remediation of migrating contaminant plumes in groundwater (Obiri-Nyarko et al., 2014). Other interventions aim at preventing the migration of contaminants without their removal, such as cement encapsulation (Álvarez-Ayuso et al., 2022). Another remediation technique consists in landfill mining, i.e., the extraction of materials or other solid natural resources from waste that has been previously disposed of by burying them in landfills and dumpsites. It has been implemented in more than 50 sites worldwide (Wagner & Raymond, 2015). Other authors (Choudhury et al., 2025) have discussed mycoremediation as a promising bioremediation strategy that could be implemented for the remediation of soil contaminated with emerging contaminants such as pharmaceuticals and personal care products, although advanced research on the topic is still necessary.

Other authors (Lavagnolo et al., 2023) have proposed the semi-aerobic landfill as probably the most appropriate system in LMICs. Depending on site-specific conditions, it may be also implemented in existing dumpsites, although significant efforts must be taken into account. The system relies on passive air ventilation, without the use of advanced technological equipment, to enhance oxygen penetration into the waste mass through natural advection processes. This promotes accelerated waste stabilization and facilitates nitrogen removal from leachate.

However, additional long-term strategies focused more on prevention should also be implemented. They should be based on the circular economy approach, considering the concept of appropriate technologies which can be defined as: a strategic approach that empowers individuals to overcome poverty and enhance their economic status by fulfilling their basic needs (Murphy et al., 2009). This is achieved through the development of their skills and capabilities, utilizing available resources in an environmentally sustainable manner. The appropriate technologies concept encompasses both “hard” and “soft” aspects of technology, including not only physical tools but also mechanisms for knowledge transfer, capacity building, and communication. Additionally, it considers the social, cultural, and gender implications of technology implementation.

The circular economy approach is essential and should be born in mind as a long-term solution, focusing on transforming end-of-life materials into resources to minimize waste generation. Objects should be reused as much as possible, repaired when broken, and recycled when reuse is not feasible (Stahel, 2016). This approach reduces the demand for virgin materials, extends the lifespan of landfills and dumpsites, and decreases the need for new waste disposal sites. Focusing on the organic fraction of solid waste will be pivotal, as it typically constitutes the majority, often exceeding 50% of MSW in LMICs (Kaza et al., 2018). Various treatment technologies are suitable to create valuable products from biowaste (Lohri et al., 2017). Another waste minimization strategy involves banning single-use plastics, which has proven successful in countries like Rwanda and Kenya (Behuria, 2021). Virgin plastics (excluding bioplastics) are typically produced from fossil fuels such as oil and natural gas; thus, uncontrolled burning of plastics disposed of in dumpsites contributes to GHG emissions from fossil fuels sources (Grosso, 2022). It is associated with the release of hazardous and persistent organic pollutants such as dioxins (Velis & Cook, 2021). Thus, it is crucial to enhance plastic circularity.

Separately collecting the organic fraction of MSW and treating it through decentralized composting (e.g., windrow composting) is likely the best solution; otherwise, in some cases, composting bins at the household level would be even better. Research by Yeo and colleagues (2020) supports the decentralized composting approach. In addition, composting bins at the household level can be used to generate a mature compost and significantly reduce the costs associated with waste collection (Mahapatra et al., 2022; Vinti, 2021). Furthermore, composting is one example of how improved SWM can reduce GHG emissions (Hoy et al., 2023). While anaerobic degradation of biowaste in disposal sites produces GHGs, particularly methane, composting is instead based on aerobic degradation and primarily generates CO_2_, resulting in significantly lower GHG emissions (Yeo et al., 2020). Chowdhury et al. (2024) have recently investigated the impact of earthworms on the microbial community and functional diversity during the vermicomposting of lignocellulosic waste. The authors found that compared to composting, vermicomposting led up to a 3.5 fold increase in nutrients (nitrogen, phosphate and potassium) content, enhanced microbial biomass and respiration. However, it is crucial to implement composting with source-separated waste collection to ensure high-quality compost and reduce contamination risks (Wei et al., 2017). This way, compost can be safely used as a hygienic organic fertilizer and soil improver, with biological stability monitored (Cosenza et al., 2018). Paul et al. (2019) investigated the use of earthworms and found that their application could significantly reduce the environmental risks associated with the mixed MSW fraction present in disposal sites. Furthermore, in recent years, several authors (Chauhan et al., 2025; Chebbi et al., 2021) have proposed the valorization of agricultural residues, such as winery and olive mill waste, for the production of rhamnolipids. Rhamnolipids are biosurfactants employed in washing treatments to remediate matrices contaminated by petroleum hydrocarbons (e.g., soil and groundwater) (Liu et al., 2021). Thus, such a circular economy approach could have further benefits in the remediation of contaminated lands including dumpsites.

As a consequence, adopting a circular economy approach offers several benefits in relation to dumpsites, including:

- reducing the risk of infectious diseases associated with the organic fraction of waste;
- lowering the concentrations of heavy metals and other pollutants in leachate originating from hazardous waste, such as WEEE;
- reducing GHG emissions;
- to promote the remediation of contaminated areas by means of biosurfactants;
- extending the operational lifespan of waste disposal sites, as they receive smaller volumes of waste annually.

Additionally, when seeking to improve SWM, policy instruments that combine a global perspective with sensitivity to local contexts are essential. As already discussed, SWM faces substantially greater challenges in LMICs, where for example collection rates are often much lower, especially in rural areas. In the absence of adequate disposal systems, a larger share of waste reaches aquatic environments (Frigo et al., 2025). Consequently, tailored, context-specific solutions are required.

Policy instruments are intended to influence the behavior of societal actors; three principal categories have been identified (Rodić & Wilson, 2017): direct regulation, economic instruments, and social instruments. They can be combined with the Wasteaware benchmark indicators (Wilson et al., 2015), which support sustainable waste management by enabling benchmarking of a city’s performance across technical and governance dimensions, facilitating intercity comparisons and monitoring developments over time in both LMICs and HICs.

European Directives are often regarded as a regulatory “gold standard,” but they may be overly ambitious for many LMICs given prevailing economic and technical constraints. Core principles, such as the waste hierarchy, should be borne in mind, as should international frameworks like the Basel Convention, whose primary aims is to minimize waste generation and to prevent the export of hazardous wastes from HICs to LMICs (UN, 1989).Additionally, when adequately supported and organized, informal recycling initiatives can generate employment opportunities, enhance local industrial competitiveness, alleviate poverty, and decrease municipal expenditures on SWM and social services (Aparcana, 2017; UNEP, 2021).

## Conclusions

The rapid increase in waste generation, especially in LMICs, has led to a heavy reliance on open dumpsites, posing severe environmental and health risks. Financial and technical constraints often hinder comprehensive risk assessments and remediation strategies. Our review underscores the necessity for a standardized comparative methodology to evaluate risks and prioritize interventions at dumpsites, building upon existing frameworks. We identified and further discussed the relative HHRA as the most practical and reliable approach. Indeed, given the limitations in data collection in LMICs, initial risk assessments can be conducted using field surveys and open-access geospatial tools. High-risk sites should be prioritized for further investigation, including groundwater quality monitoring and contamination threshold assessments. Such an approach may help optimize and more effectively direct the use of public and private funds, which are often limited, particularly in LMICs.

In parallel with risk assessment, immediate measures should be implemented to mitigate environmental and health risks. These include preventing waste burning, reducing leachate generation, and restricting site access to minimize direct human and animal exposure. Furthermore, urgent interventions are required in cases of groundwater contamination to prevent further health risks. Finally, long-term waste management strategies should embrace the principles of the circular economy taking into account the concept of appropriate technologies.

Despite the pressing need for immediate interventions, sustainable waste management in LMICs requires comprehensive policy frameworks, stakeholder engagement, and technological advancements with a long-term approach.

Future research should focus on refining the relative risk assessment methodology discussed in this manuscript and applying it to real case studies in LMICs. Indeed, the lack of such studies in these regions represents the main limitation which, however, could be readily addressed by the scientific community. Future research should also validate the SWSP approach across diverse contexts as it may represent an innovation with potentially greater impact than the HHRA, given the holistic approach underpinning it. Finally, further cost-effective mitigation strategies tailored to LMICs should be investigated. Moreover, integrating remote sensing and artificial intelligence tools could improve dumpsite identification and monitoring by regulators or environmental organizations. Notably, even in HICs, it is essential to establish a hierarchy of sites to optimize the allocation of limited economic resources for their remediation.

Ultimately, addressing dumpsite-related risks necessitates a multi-tiered approach that balances immediate remediation efforts with long-term sustainability goals. Strengthening regulatory frameworks, fostering international collaboration, and promoting innovative waste management solutions will be crucial in mitigating the adverse impacts of dumpsites while advancing global environmental and public health objectives.

## Supplementary Information

Below is the link to the electronic supplementary material.

Supplementary file1 (DOCX 43 KB)

## Data Availability

The data supporting the findings of this study are available from the corresponding author upon reasonable request. All relevant data have been included within the manuscript and its supplementary materials.
