# Supplementary material for: Prioritizing dumpsite risks and interventions: an overview and framework for action with a focus on LMICs
Source: Environ Monit Assess. 2025 Dec 3;198(1):3. doi: 10.1007/s10661-025-14842-5 (PMC12672625; doi:10.1007/s10661-025-14842-5)
Supplement: Supplementary file 1 — Supplementary file1 (DOCX 43 KB) [file 10661_2025_14842_MOESM1_ESM.docx]

***Supplementary Material for:*** Prioritizing dumpsite risks and interventions: an overview and framework for action with a focus on LMICs

Table S1 – Technical data for relative risk assessment for potentially contaminated sites (translated from (ISPRA, 2023))

| **Category** | **Factors** | **Score** | **Weight** |
| --- | --- | --- | --- |
| Surface area potentially affected by contamination | S ≤1,000 m^2^ | 2 | 1 |
|  | 1,000 m^2^ ≤ S ≤ 5,000 m^2^ | 3 |  |
|  | 5,000 m^2^ ≤ S ≤ 10,000 m^2^ | 4 |  |
|  | 10,000 m^2^ ≤ S ≤ 50,000 m^2^ | 5 |  |
|  | 50,000 m^2^ ≤ S ≤ 100,000 m^2^ | 6 |  |
|  | S > 100,000 m^2^ | 7 |  |
| Pollution event | Accidental Events - Explosions | 5 | 1 |
|  | Accidental Events - Fires: | 5 |  |
|  | Accidental Events - Spillage on Soil | 7 |  |
|  | Accidental Events - Spillage on Water | 10 |  |
|  | Accidental Events - Undefined Accidental Events | 5 |  |
|  | Poor Management - Storage of Materials and Processing Intermediates | 4 |  |
|  | Poor Management - Leakage from Tanks and Pipelines | 8 |  |
|  | Poor Management - Other (Poor Management of Materials and Networks) | 4 |  |
|  | Waste - Abandonment or Uncontrolled Waste Disposal | 4 |  |
|  | Waste - Operational and Post-Operational Waste Management | 4 |  |
|  | Waste - Other Waste | 4 |  |
|  | Historical | 6 |  |
| Matrices with contaminant presence (multiple choices possible) | Surface soil | 1 | 3 |
|  | Deep soil | 2 |  |
|  | Potential Fill Materials | 3 |  |
|  | Groundwater | 4 |  |
| Other matrices involved (multiple choices possible) | Sea and sediment | 3 | 2 |
|  | Surface waters | 3 |  |
|  | Inland water sediments | 3 |  |
|  | Interstitial gases | 4 |  |
|  | Ambient air | 2 |  |
|  | None | 0 |  |
| The release of pollutants into the surrounding matrices | Yes, with HIGH probability in one surrounding matrix | 3 | 2 |
|  | Yes, with HIGH probability in multiple surrounding matrices | 6 |  |
|  | Yes, but with LOW probability in one or more matrices | 2 |  |
|  | Not known | 2 |  |
|  | NO/No longer active | 0 |  |
| The complexity of the contamination state | Multiple carcinogenic contaminants | 10 | 2 |
|  | At least one carcinogenic contaminant (site to be characterized) | 8 |  |
|  | At least one carcinogenic contaminant (site characterized) | 7 |  |
|  | Numerous (more than 5) toxic contaminants | 5 |  |
|  | Few (up to 5) contaminants and no carcinogens (site to be characterized) | 3 |  |
|  | Few contaminants and no carcinogens (site characterized) | 2 |  |
| Detected contaminants | In the surface soil/deep soil | Without a score | |
|  | In the groundwater matrix |  |  |
| Actual land use | Agricultural | 6 | 1 |
|  | Green and residential | 4 |  |
|  | Commercial and industrial | 2 |  |
| Groundwater table level of the first aquifer | ≤ 1 m | 8 | 1.5 |
|  | 1 – 4 m | 7 |  |
|  | 4 – 10 m | 5 |  |
|  | 10 – 25 m | 3 |  |
|  | > 25 m | 1 |  |
|  | Not known | 4 |  |
|  | Absence of aquifer | 0 |  |
| Presence of artificial impermeable layers protecting the aquifer | No | 5 | 1.5 |
|  | Yes, but partial | 2 |  |
|  | Yes | 0 |  |
|  | No, but presence of impermeable lithologies (silt-clay, clay) | 0 |  |
| Site accessibility | Fenced site | 0 | 1 |
|  | Accessible site but located in a difficult-to-reach area | 2 |  |
|  | Partially fenced site | 3 |  |
|  | Accessible site | 4 |  |
| Use of water resources within the site (wells/springs) | Drinkable | 10 | 1 |
|  | Irrigation/livestock | 7 |  |
|  | Hygienic/domestic (non-drinkable) | 4 |  |
|  | Industrial | 3 |  |
|  | Not known | 4 |  |
|  | None | 0 |  |
| Distance from wells/springs and other water collection points (groundwater/surface water) intended for human consumption (domestic drinkable) | ≤100 m | 10 | 1 |
|  | 100 – 500 m | 8 |  |
|  | 500 – 1,000 m | 6 |  |
|  | 1,000 – 5,000 m | 3 |  |
|  | > 5,000 m | 0 |  |
| Distance from the surface water body/sea | ≤ 100 m | 6 | 1 |
|  | 100 – 500 m | 4 |  |
|  | 500 – 2,000 m | 2 |  |
|  | > 2,000 m | 0 |  |
| Distance from the site boundaries to the nearest inhabited center/urban agglomeration (X) or to the nearest residential areas (Y) | X ≤ 50 m | 8 | 1 |
|  | 50 m ≤ X ≤ 200 m | 7 |  |
|  | 200 m ≤ X ≤ 1,000 m | 5 |  |
|  | X > 1,000 m | 3 |  |
|  | Y ≤ 50 m | 8 |  |
|  | 50 m ≤ Y ≤ 200 m | 6 |  |
|  | 200 m ≤ Y ≤ 1,000 m | 4 |  |
|  | Y > 1,000 m | 2 |  |
| Distance from natural protection areas | Site located within natural protection areas | 8 | 1 |
|  | ≤ 200 m | 6 |  |
|  | 200 – 1,000 m | 4 |  |
|  | > 1,000 m | 0 |  |
| Site affected by hydrogeological instability *(based on the Italian legislation) | Located in a PAI area with P3/P4* hazard (high to very high landslide hazard) | 8 | 1 |
|  | Located in a PAI* area with P1/P2* hazard (moderate to medium landslide hazard) | 4 |  |
|  | Located in an area with P3* hydraulic hazard (frequent floods) | 8 |  |
|  | Located in an area with P2* hydraulic hazard (infrequent floods) | 4 |  |
|  | No | 0 |  |
| Spread of contamination outside the site | Confirmed | 8 | 2 |
|  | Probable, but not yet confirmed | 5 |  |
|  | Unlikely/No | 2 |  |
| Additional issues (multiple choices possible) | Site located in an area with confirmed epidemiological and health criticalities | 8 | 1.5 |
|  | Site adjacent (within 100 m) to a sensitive receptor | 6 |  |
|  | Site located in an area affected by widespread anthropogenic pollution | 2 |  |
|  | Site affected by probable indoor vapor intrusions or significant indoor/outdoor dust exposures | 5 |  |
|  | Site adjacent to areas used for agricultural production and/or livestock | 5 |  |
|  | Site with abandoned structures, at high static/structural risk (collapse) | 2 |  |
|  | None | 0 |  |

Table S2 – Technical data for relative risk assessment for suspected contaminated landfills and dumpsites (translated from (Sicilian Region, 2016))

| **Category** | **Factors** | **Score** |
| --- | --- | --- |
| **Source (S)** | | |
| 1 – Kind of waste | Inert | 0 |
|  | Municipal | 1 |
|  | Non-Hazardous Special / | 2 |
|  | 3 – Hazardous Special / | 3 |
|  | Not defined | 3 |
| 2 - Horizontal surface (m^2^) or volume (m^3^) occupied by waste (*to be multiplied by the score related to the kind of waste*) | < 1,000 m^2^ or < 2,000 m^3^ | 1 |
|  | 1,000 – 5,000 m^2^ or 2,000 – 10,000 m^3^ | 2 |
|  | 5,000 – 15,000 m^2^ or 10,000 – 30,000 m^3^ | 3 |
|  | ≥ 15,000 m^2^ or ≥ 30,000 m^3^ | 4 |
| 3 – Presence of leachate | Yes | 3 |
|  | No | 0 |
| 4 – Presence of biogas | Yes | 2 |
|  | No | 0 |
| 5 – Presence of asbestos | No | 0 |
|  | In sheets or wool | 1 |
|  | In fragments | 2 |
|  | In powder or No information | 3 |
| 6 - Presence of gutter channel | Yes | 0 |
|  | No | 2 |
| 7 – Surface covering | Yes | 0 |
|  | No | 2 |
| 8 - Fencing | Yes | 0 |
|  | No | 1 |
| 9 - Age of the site (evaluated with respect to the year of site closure) | ≥ 30 years | 0 |
|  | 20 – 30 years | 1 |
|  | 10 – 20 years | 2 |
|  | < 10 years or No information | 3 |
| $S_{tot}= \sum_{i=1}^{9} i$ (max = 28) | | |
| Weighting factor (W) = 1.190 | | |
| Source score = S_tot_ × W (max = 33.33) | | |
| **Vector (V)** | | |
| 10 - Substrate type/permeability of substrate | Clay or lower permeability | 0 |
|  | Silt, marl or Low permeability | 1 |
|  | Lithoid rock | 2 |
|  | Sand or gravel or higher permeability or No information | 3 |
| 11 - Presence of bottom impermeable liner | Yes | 0 |
|  | No | 2 |
| 12 – Groundwater table level | ≥ 40 m | 0 |
|  | 20 – 40 m | 1 |
|  | 5 - 20 m | 2 |
|  | < 5 m or no information | 3 |
| 13 - Proximity to surface water bodies | ≥ 150 m | 0 |
|  | 100 – 150 m | 1 |
|  | 50 – 100 m | 2 |
|  | < 50 m or no information | 3 |
| 14 – Leachate collection system | Yes | 0 |
|  | No | 3 |
| 15 – Biogas collection system | Yes | 0 |
|  | No | 2 |
| 16 - Presence of landslides | Yes | 3 |
|  | No | 0 |
| $V_{tot}= \sum_{i=10}^{16} i$ (max = 19) | | |
| Weighting factor (W) = 1.754 | | |
| Vector score = V_tot_ × W (max = 33.33) | | |
| **Target (T)** | | |
| 17 – Proximity of wells | ≥ 500 m or no wells | 0 |
|  | 200 – 500 m | 1 |
|  | 50 – 200 m | 2 |
|  | < 50 m | 3 |
| 18 – Type of well (*to be multiplied by the score related to the proximity of wells*) | Drinkable | 2 |
|  | For irrigation purposes | 1 |
|  | For industrial purposes | 0.5 |
| 19 – Type of area | Industrial area or uncultivated area | 0 |
|  | Road infrastructures | 1 |
|  | Agricultural, commercial or artisanal area | 2 |
|  | Protected natural area, water body, residential area or no information | 3 |
| 20 – Proximity to agricultural activities | No or ≥ 500 m | 0 |
|  | 200 – 500 m | 1 |
|  | 50 – 200 m | 2 |
|  | < 50 m or no information | 3 |
| 21 – Proximity to livestock farms | No or ≥ 500 m | 0 |
|  | 200 – 500 m | 1 |
|  | 50 – 200 m | 2 |
|  | < 50 m or no information | 3 |
| 22 – Proximity to inhabited areas | No or ≥ 1,000 m | 0 |
|  | 200 – 1,000 m | 1 |
|  | 50 – 200 m | 2 |
|  | < 50 m or no information | 3 |
| 23 – Proximity to isolated houses | No or ≥ 500 m | 0 |
|  | 200 – 500 m | 1 |
|  | 50 – 200 m | 2 |
|  | < 50 m or no information | 3 |
| 24 – Proximity to surface water bodie | No or ≥ 500 m | 0 |
|  | 200 – 500 m | 1 |
|  | 50 – 200 m | 2 |
|  | < 50 m or no information | 3 |
| 25 – Proximity to valuable areas | No or ≥ 500 m | 0 |
|  | 200 – 500 m | 1 |
|  | 50 – 200 m or presence of regulatory constraints | 2 |
|  | < 50 m or no information | 3 |
| 26 – Proximity to the water network | No or ≥ 500 m | 0 |
|  | 200 – 500 m | 1 |
|  | 50 – 200 m | 2 |
|  | < 50 m or no information | 3 |
| $V_{tot}= \sum_{i=26}^{17} i$ (max = 30) | | |
| Weighting factor (W) = 1.111 | | |
| Target score = T_tot_ × W (max = 33.33) | | |
| **Overall site score = S + V + T (max = 100.00)** | | |

**References**

ISPRA, 2023. Strumenti per la sperimentazione dei criteri nazionali di priorità d’intervento nei siti potenzialmente contaminati. Rome, Italy.

Sicilian Region, 2016. Update of the regional remediation plan - Part 2 (in Italian).
